# Conversion surgery for undifferentiated carcinoma with osteoclast-like giant cells of the pancreas: a case report

**DOI:** 10.1186/s40792-022-01385-x

**Published:** 2022-03-14

**Authors:** Yosuke Igarashi, Takeshi Gocho, Tomohiko Taniai, Tadashi Uwagawa, Ryoga Hamura, Yoshihiro Shirai, Jungo Yasuda, Koichiro Haruki, Kenei Furukawa, Toru Ikegami

**Affiliations:** Division of Hepatobiliary and Pancreas, The Jikei University School of Medicine, 3-25-8, Nishi-Shinbashi, Minato-ku, Tokyo, 105-8461 Japan

**Keywords:** Undifferentiated carcinoma with osteoclast-like giant cells, Conversion surgery, Pancreatic ductal adenocarcinoma, FOLFIRINOX

## Abstract

**Background:**

Undifferentiated carcinoma with osteoclast-like giant cells (UCOGCs) is a rare subtype of pancreatic cancer (PC), and its clinicopathological characteristics are still unclear. Herein, we report a case of initially unresectable UCOGC that was successfully resected after FOLFIRINOX therapy.

**Case presentation:**

A 63-year-old man was referred to us for evaluation of a pancreatic mass detected by computed tomography (CT) during a medical checkup. Computed tomography showed a 7.5-cm tumor located in the pancreatic head and body, which involved the common hepatic artery (CHA), gastroduodenal artery (GDA), and main portal vein (PV) with tumor thrombus. UCOGC was suspected by endoscopic ultrasonography-guided fine needle aspiration, and the patient was diagnosed with unresectable locally advanced pancreatic cancer. After ten cycles of FOLFIRINOX, the tumor size decreased to 3 cm and the tumor thrombus in the main portal trunk had disappeared in the follow-up CT scan. However, the patient experienced severe adverse drug reactions, including neutropenia and liver dysfunction. Therefore, we performed pancreatoduodenectomy with portal vein resection. The pathological diagnosis was UCOGC with a negative tumor margin. He was treated with FOLFIRINOX, and remains recurrence-free for 6 months after surgery.

**Conclusions:**

We experienced a case undergoing conversion surgery for unresectable UCOGC, which resulted in R0 resection. FOLFIRINOX could be a possible regimen to achieve conversion surgery for UCOGC.

## Background

Pancreatic cancer (PC) is a gastrointestinal cancer with a poor prognosis and limited potential for oncologic treatment. Undifferentiated carcinoma with osteoclast-like giant cells (UCOGCs) is a rare subtype of pancreatic ductal adenocarcinoma (PDAC), which accounts for less than 1% of non-endocrine pancreatic tumors [1]. Prognosis for patients with UCOGCis inconsistent relative to general PDAC [1–4], and the clinicopathological characteristics of UCOGC have not been elucidated. Herein, we experienced a case of initially unresectable UCOGC who underwent conversion surgery.

## Case presentation

**Fig. 1:**
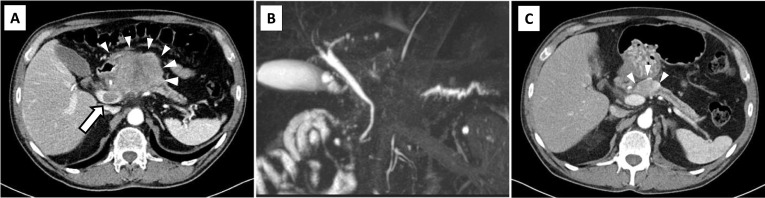
Computed tomography showed the pancreatic mass which was 7.5 cm in size at initial diagnosis (**A**, arrowhead). It revealed portal vein embolism (**A**, arrow), which was suggestive of tumor emboli at the main trunk and stenosis of the main pancreatic ducts with distal dilatation of the pancreatic duct (**A**). Magnetic resonance cholangiopancreatography exhibited the main pancreatic duct (MPD) stenosis at the pancreatic head with distal dilatation of MPD (**B**). After ten cycles of chemotherapy with FOLFIRINOX, the tumor shrunk to 3 cm (**C**, arrowhead), and the thrombi in the main portal vein could not be identified (**C**)

A 63-year-old man was admitted to our hospital for further evaluation of an asymptomatic pancreatic mass that was detected by computed tomography (CT) during a medical checkup. He had a medical history of hypertension and was treated with 20 mg of daily olmesartan medoxomil. Laboratory data showed a slightly elevated hemoglobin A1c of 6.8% and tumor markers were as follows: carcinoembryonic antigen (CEA), 3.7 ng/mL; cancer antigen-19–9 (CA19-9), 147 U/mL; cancer antigen-125, 42 U/mL; DUPAN-2, 120 U/mL; and Span-1, 72 U/m. A CT scan revealed a 7.5-cm mass located in the pancreatic head and body (Fig. 1A) that invaded the common hepatic artery (CHA), gastroduodenal artery (GDA), and proper hepatic artery (PHA). A tumor thrombus in the main portal vein (PV) was also detected. The celiac artery (CA) and superior mesenteric artery (SMA) were intact. Magnetic resonance cholangiopancreatography (MRCP) revealed main pancreatic duct (MPD) stenosis at the pancreatic head with distal dilatation of the MPD (Fig. 1B). Endoscopic ultrasonography-guided fine needle aspiration was performed, and UCOGC was suspected based on pathology. Therefore, the patient was diagnosed with unresectable locally advanced UCOGC and conventional FOLFIRINOX (oxaliplatin, 85 mg per square meter of body-surface area; irinotecan, 180 mg per square meter; leucovorin, 400 mg per square meter; and fluorouracil, 400 mg per square meter given as a bolus followed by 2,400 mg per square meter given as a 46-h continuous infusion, every 2 weeks) was administered. After 10 cycles (20 weeks) of FOLFIRINOX, contrast-enhanced CT showed a significant response to chemotherapy. Although the tumor was still in contact with the CHA and GDA, the tumor diameter shrank to 3 cm and the portal vein thrombus had disappeared (Fig. 1C). The tumor markers were reduced as follows: CEA, 2.3 ng/mL; CA 19–9, 18 U/mL; DUPAN-2, 25 U/mL. Severe adverse drug reactions including neutropenia and liver dysfunction were also observed, and it was difficult to continue FOLFIRINOX therapy. Therefore, a conversion surgery was performed.

**Fig. 2:**
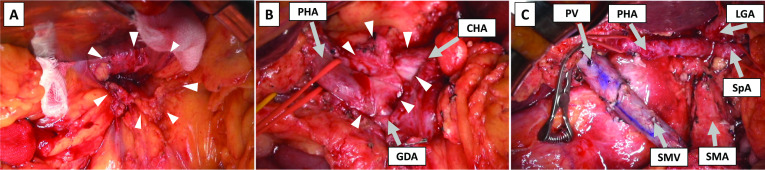
Intraoperative findings showed the tumor invading the transverse mesocolon. White arrows indicate tumor invasion to the transverse mesocolon (**A**). The tumor invasion was suspected at the neural plexus around the common hepatic artery and gastroduodenal artery. White arrows indicate tumor invasion to the neural plexus around the hepatic arteries (**B**). Complete tumor resection was achieved by resecting the neural plexuses and superior mesenteric vein (SMV) with 4 cm length. White arrows indicate remaining tumor cells surrounded by fibrosis (**C**)

Intraoperative findings showed no peritoneal dissemination or liver metastasis. The tumor invaded the transverse mesocolon (Fig. 2A), but the SMA was not affected. Although tumor invasion to the neural plexus around the CHA and GDA was suspected (Fig. 2B), complete resection of these plexuses was achieved. After confirming no tumor invasion to the plexus by examining the intraoperative frozen section, the pancreatic parenchyma was dissected with a 3-cm tumor margin. Since PV invasion was strongly suspected, the PV/superior mesenteric vein (SMV) was resected 4 cm in length and reconstructed with end-to-end anastomosis. Finally, the pancreaticoduodenectomy with Child’s reconstruction was performed. The total operation time was 597 min, and the blood loss was 1,120 mL.

**Fig. 3:**
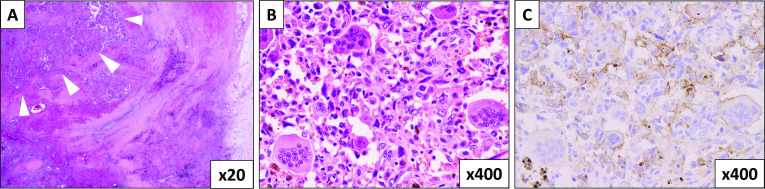
Histopathological findings showed that the peritumoral area is highly fibrotic, which suggested that the tumor had shrunk by FOLFIRINOX. White arrows indicate remaining tumor cells surrounded by fibrosis (**A**) (HE × 20). The tumor cells were pleomorphic, irregularly shaped to spindle-shaped, and surrounded by osteoclast-like multinucleated giant cells, consistent with the diagnosis of UCOGC (**B**), (HE × 400). Both the cytoplasm of the cancer cells and osteoclast-like giant cells are positive for PD-L1 immunostaining (**C**) (PD-L1 immunostaining × 400)

The excised tumor was 3.5 × 3.1 × 2.4 cm in size and was located across the pancreatic head and body. Histopathological findings showed that the tumor cells were pleomorphic, irregularly shaped to spindle-shaped, and surrounded by osteoclast-like multinucleated giant cells, which was consistent with the diagnosis of UCOGC (Fig. 3A, B). Immunohistochemistry (IHC) showed positive tumor staining for programmed death-ligand 1 (PD-L1) (Fig. 3C). Some adenocarcinoma components were also identified. Tumor cell clusters were found in the portal vein. Tumor metastasis was found in one of the #8 lymph nodes in the anterosuperior region along the CHA, out of all 26 dissected nodes. The UICC TNM classification (8th edition) was defined as ypT2N1M0, stage IIB. No tumor exposure of the margins was observed; therefore, histological R0 was achieved. The postoperative course was uneventful, and the patient was discharged on the 16th postoperative day. He is now receiving adjuvant FOLFIRINOX without recurrence for more than 6 months after the operation.

## Discussion

We successfully treated an unresectable locally advanced UCOGC case with conversion surgery after FOLFIRINOX therapy. PC is the most aggressive solid tumor in humans and has a very poor prognosis [5, 6]. Even if no metastasis is detected, it is often diagnosed as unresectable locally advanced pancreatic cancer (LAPC); the primary resectable PC is present in only 15–20% of all patients [7]. However, the number of cases of conversion surgery has been increasing due to the development of novel chemotherapeutic regimens. Of these regimens, FOLFIRINOX has been reported to improve the response rate of LAPC and the rate of conversion surgery considerably [7, 8]. To the best of our knowledge, there have been no reported cases of conversion surgery for UCOGC after FOLFIRINOX, and the current case is the first report.

UCOGC is extremely rare, with an incidence of 1.4% for invasive PCs and 0.4% for resected PCs [2]. The prognosis of UCOGC is inconsistent, because the disease is so rare that it is difficult to evaluate the prognosis by stage [1], and most UGOCG cases were found at the advanced stage and recurred early after resection [9]. Another reason is that UCOGC was not defined until 2010 when WHO integrated two subtypes, giant cell tumors (GCTs) with osteoclast-like cells and pleomorphic GCTs, as UCOGC [10]. In general, UCOGC is a rapidly growing tumor with abundant blood flow, often presenting as a large tumor with hemorrhage and necrosis [11, 12]. UCOGC is also an aggressive tumor, so composite resection with invaded adjacent organs such as the stomach, jejunum, colon, left kidney [13], diaphragm, and common hepatic artery is often needed [14]. Further investigations with large sample sizes are required to identify the molecular characteristics of UCOGC and establish a treatment strategy for UCOGC to achieve conversion surgery.

Conversion surgery may improve the prognosis of patients with PC. The number of cases with conversion therapy for PDAC has been increasing, owing to the recent advances in chemotherapy [6]. Although the efficacy of chemotherapy for UCOGC has not yet been fully evaluated, standard chemotherapy regimens for pancreatic cancer, including FOLFILINOX, have been selected because UCOGC is considered a variant of ductal adenocarcinoma of the pancreas [9]. In addition to FOLFIRINOX, there are reports that gemcitabine was effective against UCOGC [15]. Gemcitabine is often used in combination with nab-paclitaxel, capecitabine, or S-1. In addition to conventional chemotherapy, immune checkpoint inhibitors (ICIs) have recently attracted increasing attention for the treatment of various cancers, including PC [5]. IHC for PD-L1 was positive in 65% to 80% of UCOGC cases, and these cases showed poor prognosis [16, 17]. However, PD-L1-positive UCOGC showed a marked response to ICI, both in primary UCOGC tumors and metastatic diseases [18]. In the current case, IHC for PD-L1 was strongly positive in the surgically resected specimen, suggesting that ICIs may have been effective. Routine assessment of immune-related markers such as programmed death 1 (PD-1) and PD-L1 for tumor biopsy specimens at initial diagnosis could provide therapeutic options other than FOLFIRINOX to establish precision treatment for PC in the era of immunotherapy. Another important examination for patients with PDAC is germline genetic testing for BRCA pathogenic variant. For patients who have germline BRCA mutation, after at least 16 weeks of platinum-based chemotherapy such as FOLFIRINOX without disease progression, the PARP inhibitor olaparib is recommended [19]. Although olaparib is usually used in the second-line setting, we consider BRCA status of the patient in the current study is very important because the patient could be susceptible to tumor recurrence after conversion surgery. Since the patient underwent 20 weeks of preoperative FOLFIRINOX with favorable response, we plan to check BRCA status in the near future.

## Conclusions

We encountered a case of conversion surgery for UCOGC of UR-LAPC, which resulted in R0 resection. Administering a FOLFIRINOX regimen can create more favorable conditions to perform conversion surgery for unresectable UCOGC. Moreover, routine assessment of immune-related markers may be useful for establishing precision medicine in patients with PC.

## Data Availability

Not applicable.

## Abbreviations

PC: Pancreatic cancer
UCOGC: Undifferentiated carcinoma with osteoclast-like giant cells
PDAC: Pancreatic ductal adenocarcinoma
CT: Computed tomography
CHA: Common hepatic artery
GDA: Gastroduodenal artery
PHA: Proper hepatic artery
PV: Portal vein
CA: Celiac artery
SMA: Superior mesenteric artery
MRCP: Magnetic resonance cholangiopancreatography
MPD: Main pancreatic duct
SMV: Superior mesenteric vein
IHC: Immunohistochemistry
PD-L1: Programmed death-ligand 1
LAPC: Giant cell tumors
GCTs: Locally advanced pancreatic cancer
GEM: Gemcitabine
nab-PTX: Nanoparticle albumin-bound paclitaxel
ICI: Immune checkpoint inhibitor
PD-1: Programmed death 1
